# A dual limb attention based deep learning network for multipath classification of millimetre wave signals in intelligent transportation system

**DOI:** 10.1038/s41598-026-39131-0

**Published:** 2026-03-05

**Authors:** Aathira G. Menon, Prabu Krishnan, Shyam Lal, Shilpa Suresh

**Affiliations:** 1 Department of Electronics and Communication Engineering, National Institute of Technology Karnataka, Surathkal, Mangalore, 575025 India; 2 Manipal Institute of Technology, Manipal Academy of Higher Education, Manipal, India

**Keywords:** Multipath, mm-wave, Intelligent transportation system, CNN, Attention, Engineering, Mathematics and computing

## Abstract

The presence of line-of-sight (LOS) and non-line-of-sight conditions in an intelligent transportation system (ITS) is critical, as it directly impacts the localisation and overall system performance. Distinguishing LOS, first-order and higher-order multipaths (HOMP) is hindered by the dense multipath (MP) propagation. The computational resource requirement and limited accuracy associated with traditional iterative methods have stimulated the use of deep learning (DL). This work proposes a novel, lightweight, dual limb attention (DLA) based DL network termed as “MOVENetx64”, for robust identification of MPs in ITS. DLA mechanism stacked with convolutional neural networks and long-short-term memory layers forms the prominent feature extractor, which enables feeding raw, unprocessed data into the DL pipeline. A novel alternating loss strategy is designed to nullify the effect of the extreme imbalance associated with the target output classes. Ray-tracing-based vehicular datasets for City, Suburban, and Highway are generated. A combination of time, received power, angular characteristics, and phase spectrum forms the input feature-set. The proposed MOVENetx64 showcases MP classification accuracy of (98.71%,99.41%,96.44%), and achieves the least HOMP prediction error of (0.6%,0.17%,2.28%) on the datasets. These results validate that the proposed MOVENetx64 is scalable and computationally efficient for distinguishing multipaths efficiently and enabling reliable vehicular communication in ITS.

## Introduction

Next-generation vehicular technology is envisioned for high-frequency technologies, such as millimetre-wave (mm-wave) and terahertz (THz)^1^. Nevertheless, these systems are affected by high propagation losses (40 dB) because of obstruction by buildings, vehicles, road surfaces, and other geographical obstructions that may impede propagation of a direct line-of-sight (LOS) signal^1^. These obstacles also cause reflection, scattering, and diffraction, which leads to the generation of multiple delayed replicas of the signal, which are termed as multipath components (MPCs).

Each MPC arrives at the receiver at slightly different times and in slightly different directions. Higher-order multipath (HOMP) imposes temporal, spatial, and angular distortions, introducing interference, prolonged delay-spread, and ambiguities in time-of-arrival (ToA), angle-of-arrival (AoA), and Doppler estimation, all of which are critical to vehicular communications. These impairments deteriorate the performance of vital applications, such as autonomous navigation^2^, search and rescue, real-time vehicle surveillance^3^, parking space estimation^4^, crowd-sensing^2^, and localisation accuracy. In a nutshell, multipath can degrade the reliability and performance of intelligent transportation systems (ITS)^5,6^.

MPC effects are amplified in dynamic, time-varying, and non-stationary vehicular environments^7^. Constructive interference may boost the signal, whereas destructive interference may corrupt the signal, causing depression points^8^. At depression points, the signal strength can be less than the sensitivity level of the receiver, causing a loss of connection or an increase in bit error rates. Due to the rich-scattering environment, direct LOS communication is rarely possible in a real-world vehicular scenario. Therefore, it is essential to consider the first-order multipath (FOMP) along with LOS as dominating links, while eliminating the traces of HOMP.

Traditional multipath classification methods employ mathematical modelling and iterative methods^9–11^ to identify NLOS/MP using a deterministic threshold. Due to the dynamic and non-stationary nature of vehicular networks, it is seldom possible to extract an accurate threshold^12^, which compromises the accuracy of detecting multipaths. The massive expense associated with lab upfront, sophisticated hardware requirements, and prolonged testing time hinders the performance of these techniques^11^. Also, these methods can attain a limited accuracy up to $\approx$90%, which is insufficient for a real-time, dynamic vehicular network^9,10^. In a real-time ITS, the inter-symbol interference and the overlapping of signal properties of different MPCs^13^, along with the highly time-varying channels^14^ and dense multipath propagation^15^, complicate the process of identifying the type of MPs. The extreme imbalance in the density of HOMP and that of the direct links/FOMPs further complicates the MP classification.

In order to combat the above concerns, artificial intelligence (AI)-based solutions have gained popularity due to their effective feature extraction, superior data-handling and quick response compared to traditional methods^11,16,17^. Alongside AI, it is also essential to choose key parameters to achieve the optimal MP classification. In^1^, authors have addressed a non-AI-based solution for LOS analysis by considering the location, height and density of the blockages. In contrast, the authors in^18–21^emphasised that channel impulse response (CIR), time and direction-based features are often more significant than the physical characteristics of obstacles. In^7,22–28^, authors have solely utilised CIR information to estimate the MP. However, in real-time, it is highly expensive to extract CIR. Also, the noise associated with the reconstructed CIR could degrade the overall system performance^29^. Hence, time and direction-based features are considered as the potential parameters for efficiently identifying MP, as presented in^25,30,31^.

To achieve a promising feature extraction, the authors in^22^ have transformed 1D feature set into 2D image tensor. Although this approach can improve the feature extraction, it also introduces redundant complexity due to the 2D feature set^32^. Additionally, frequency-based detection can result in the potential loss of time-correlated features^33^. Thus, using 1D time-series data is often more effective. Advanced DL models like convolutional neural networks (CNN), deep neural networks (DNN), long short-term memory (LSTM) and generative adversarial networks (GAN) can analyse 1D feature sets effectively, as they extract spatial and temporal features, overcome feed-forward issues and capture sequential dependencies^11,34^. The studies referenced in^35–39^ ascertain the improved performance achieved with the use of these powerful models. An ensemble of these models, along with special transformations like Morlet-wavelet transform (MWT)^36^, can uplift the feature extraction and thereby promise a precise removal of MP as highlighted in^25,40–43^. Also, MWT requires extra time and resources to implement the transformations. However, many of these methods focus primarily on indoor systems, and MWT requires additional time and resources.

To identify and classify the MP at a granular level, recent studies referenced as^11,26,37,44,45^considered a multi-class classification problem to mitigate the MP. However, an increased number of classes can introduce a threshold overlap and increased resource requirements, which can cause a negative impact on the classification^46^. Hence, it is recommended to retain a minimal number of target classes. Though most of the recent literature on MP classification is dedicated to indoor localisation, limited attention is paid to terrestrial diverse vehicular scenarios. In^27^, although the study considered three diverse driving scenarios to identify the type of MP, the dynamic characteristics of the driving environments were overlooked, which have been incorporated in^7^ for an indoor communication setup. Table 1 compares key parameters of existing multipath classification techniques with the proposed framework.

Though there exists plenty of research on multipath classification, no prior study is focused on developing a powerful feature extractor dedicated to 1D sequential data. To address these concerns, this paper focuses on developing a novel, intelligent multipath classification framework called *MOVENetx64* having a novel and efficient feature extractor block. The principal contributions of this study to the literature are as follows:

1. MOVENetx64-a new, lightweight DL-based classifier is proposed, having a novel feature extractor block termed as Dual Limb Attention (DLA), which is designed specifically for 1D sequential data. MOVENetx64, equipped with DLA, will be capable of handling raw, unprocessed data, and thereby slash the heavy duration associated with data preparation and processing.
2. A novel loss function termed as Alternating Loss Strategy (ALS) is developed to nullify the effect of extreme class imbalance between the target classes to enable optimal training.
3. Three near-real-time diverse use-cases on City, Suburban and Highway vehicular networks are generated using the Ray Tracing technique to train and evaluate the proposed MOVENetx64. The effects of the Doppler shift and geographical obstacles are introduced to emulate the intricacies of the Indian vehicular scenario, validating the adaptability of the proposed model to perform effectively in diverse use cases.
4. Under each vehicular use-case, a rich combination of time, power, angular characteristics and phase spectrum is chosen as the feature set to capture all the prime information about the multipaths.
5. An improvement of 84.69%, 77.92% and 50.96% on HOMP prediction error across the City, Suburban and Highway scenarios, respectively, is achieved by the proposed MOVENetx64 in comparison to the state-of-the-art DL classifiers.
6. The proposed MOVENetx64 exhibits promising performance on diverse vehicular datasets, which justifies the versatility of the proposed model in adapting to various driving scenarios.

The rest of this article is structured as follows: The system model and the proposed MOVENetx64 architecture are detailed in the “Proposed system” section. The “Training and implementation” section focuses on the training and implementation details of the proposed framework. The experimental outcomes of the proposed model and the comparative study with the respective state-of-the-art models are discussed in “Results and discussion” section. Finally, the paper is concluded.

**Table 1:** Comparison of existing multipath classification solutions against the proposed framework.

		Driving scenario			Features									
Reference	Region of operation	C	S	H	Power	Phase	Time	Angle	CIR	Delay spread	Model	Class	Dynamic	Doppler effect
^47^ (2023)	Outdoor	✓	✗	✗	✓	✗	✓	✓	✗	✗	ML	Binary	✗	✗
^24^ (2024)	Indoor	✗	✗	✗	✗	✗	✗	✗	✓	✗	ML	Multi	✓	✗
^11^ (2024)	Outdoor	✓	✗	✗	✓	✗	✗	✗	✓	✗	ML, LSTM	Multi	✗	✗
^37^ (2024)	Outdoor	✗	✗	✓	✗	✗	✗	✗	✓	✗	CNN, LSTM	Multi	✗	✗
^30^ (2024)	Indoor	✗	✗	✗	✗	✗	✗	✗	✓	✗	ML	Multi	✗	✗
^31^ (2024)	Outdoor	✓	✗	✗	✓	✗	✗	✓	✗	✗	ANN	Binary	✗	✗
^25^ (2024)	Indoor	✗	✗	✗	✗	✗	✗	✗	✓	✗	CNN-LSTM	Binary	✗	✗
^45^ (2024)	Indoor	✗	✗	✗	✗	✗	✗	✗	✓	✗	DNN	Multi	✗	✗
^28^ (2024)	Indoor	✗	✗	✗	✗	✗	✗	✗	✓	✗	CNN-BiLSTM-TL	Binary	✗	✗
^40^ (2024)	Outdoor	✗	✓	✗	✓	✗	✓	✓	✗	✗	CNN, SVM	Multi	✗	✗
^44^ (2025)	Indoor	✗	✗	✗	✗	✗	✗	✗	✓	✗	ADCNN-BiLSTM	Binary	✗	✗
**MOVENetx64(Proposed)**	**Outdoor**	**✓**	**✓**	**✓**	**✓**	**✓**	**✓**	**✓**	✗	✗	**CNN-LSTM-Att**	**Binary**	**✓**	**✓**

Significant values are in bold.

## Proposed system

### System model

**Fig. 1:**
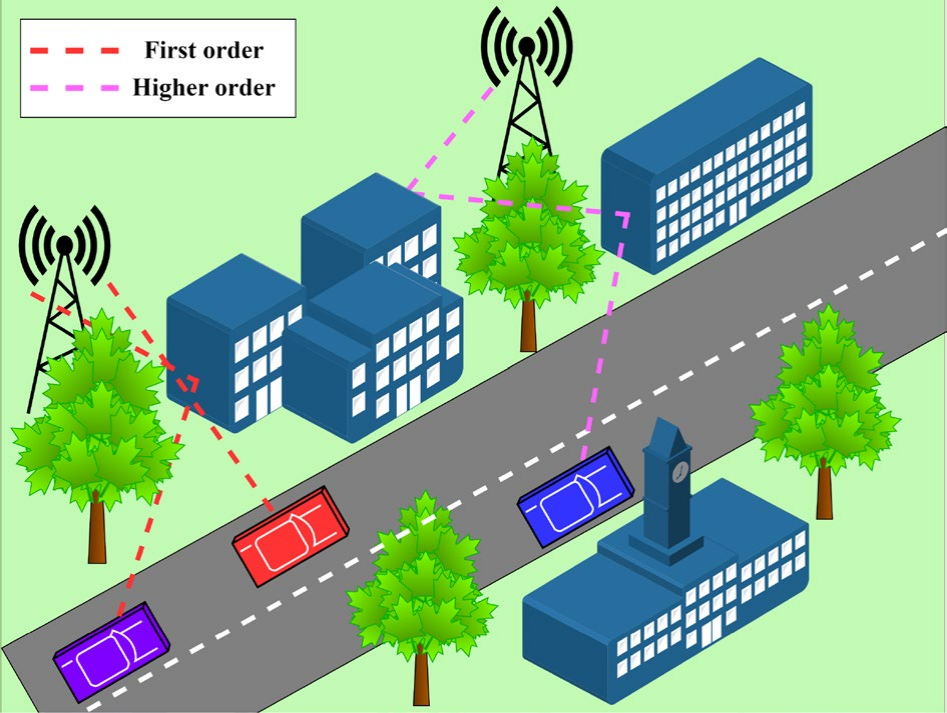
A multipath scenario in intelligent transportation system.

A typical scenario of a vehicular network is depicted in Fig. 1. Due to geographical obstacles, such as tall buildings and tree canopies, the direct link between the base station (BS) and the mobile user (MU) is obstructed, causing the rise of multipaths. These MPs can be broadly grouped into FOMP and HOMP based on their scattering/reflection/diffraction degree. HOMPs pose a significant challenge, as they induce potential noise into the system. Hence, identifying FOMPs among HOMPs is crucial to ensure the smooth functioning of a vehicular network.

Let h(t) be the channel impulse response which contains the delay ($\tau$), power ($\alpha$) and azimuth AoA ($\varphi _R$), azimuth AoD ($\varphi _T$), zenith AoA ($\Theta _R$) and zenith AoD ($\Theta _T$) based information of the m^th^ MP. The mathematical representation of h(t) with the MPC is described in Eq. 1. $\delta (.)$ is the Dirac-delta function.

1 $$\begin{aligned} \mathrm {h \big (t, \tau , \Theta , \varphi \big )} = \mathrm {\sum _{m=1}^{M_{n}} \alpha _{m} e^{j \varphi _{m}} \times \delta \big (t- \tau _{m}\big ) \times \delta \big ( \Theta _{R} - \Theta _{R,m}\big ) \times } \mathrm {\delta \big ( \Theta _{T} - \Theta _{T,m}\big )\ \times \delta \big ( \varphi _{R} - \varphi _{R,m}\big ) \times } \mathrm {\delta \big ( \varphi _{T} - \varphi _{T,m}\big )} \end{aligned}$$

### Proposed MOVENetx64

**Fig. 2:**
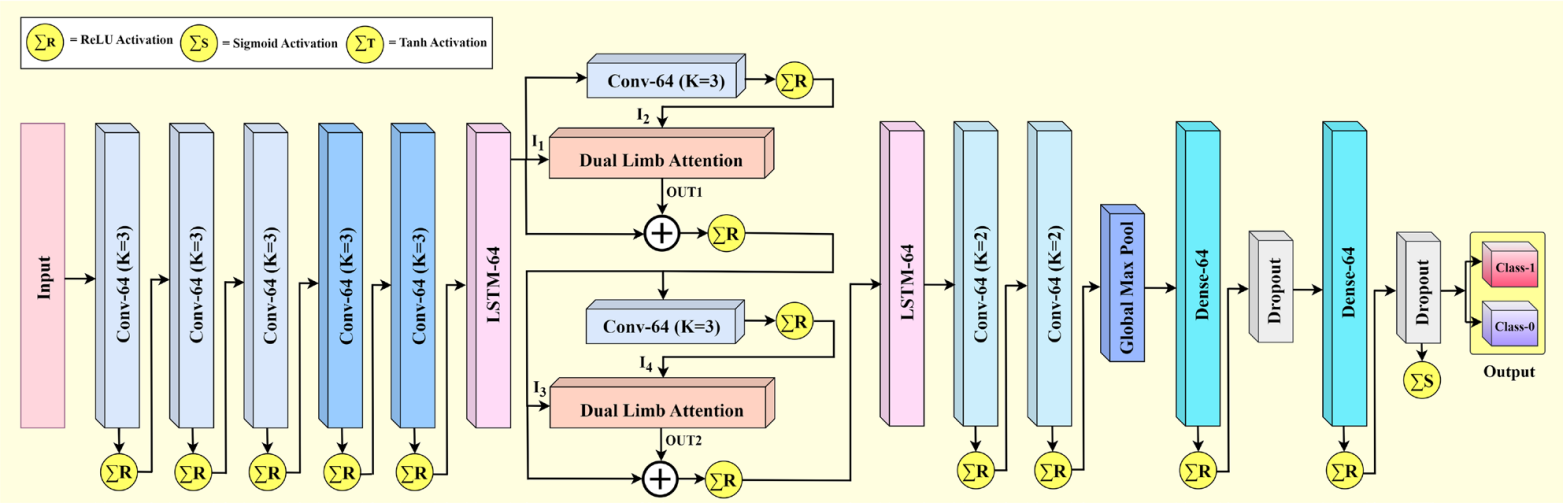
Architecture of the proposed MOVENetx64.

**Fig. 3:**
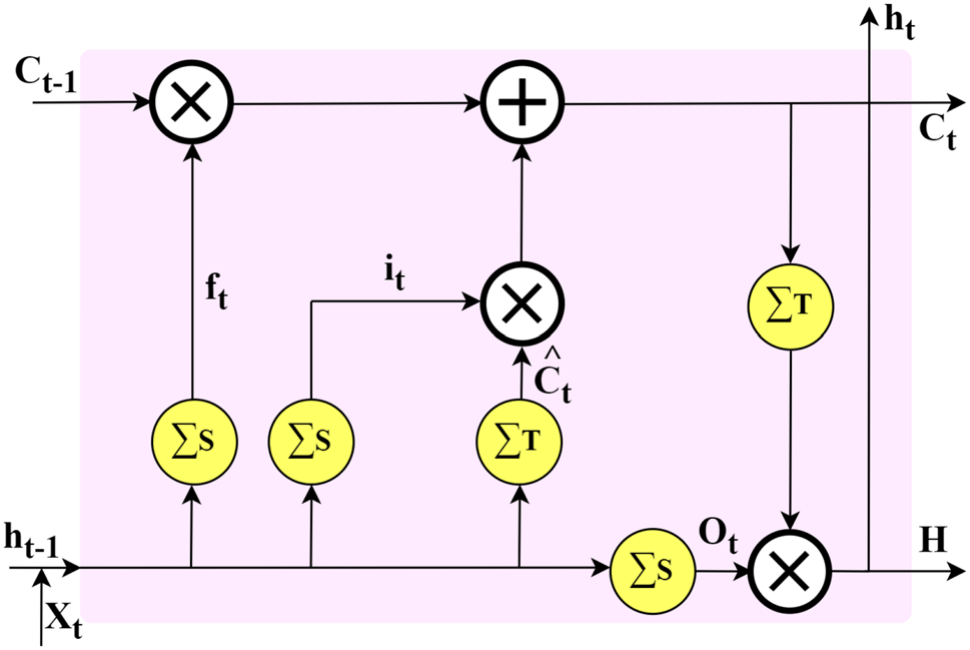
Structure of an LSTM layer.

This subsection presents the detailed mathematical analysis of the proposed MOVENetx64 model for distinguishing the multipath of mm-wave signals in an Intelligent Transportation System. MOVENetx64 is a lightweight, novel deep learning-based architecture designed using a novel attention mechanism, CNN and LSTM layers. Fig. 2 depicts the architecture of MOVENetx64, highlighting its structure, which consists mainly of three blocks: the CNN, LSTM, and a novel attention mechanism termed as Dual Limb Attention (DLA). “Dual limb Attention” section describes the novel DLA. The raw input vector ($\vec {\textrm{X}}$) is fed into a triple stack of CNN layers, which performs the feature extraction on an initial level. Due to the gating mechanism of LSTM^23^ as shown in Fig. 3, the feature extraction is thus tuned, as LSTM takes into account the memory-based information and filters out noise, which is crucial for sequential data. The mathematical abstraction of the CNN-LSTM stack is detailed in Eqs. 2 to 10.

The input $\vec {\textrm{X}}$ is fed into the three stack of 1D-CNN with ReLU ($\Phi _{R}$) activation function as presented in Eq. 2.

2 $$\begin{aligned} \mathrm {X^{(l)}_{t}} = \mathrm {\Phi _{R} \big (\sum _{m=1}^k {W_m}^{(l)} \big \{\Phi _{R} \big (\sum _{j=1}^k {W_j}^{(l)} \big [\Phi _{R} \big ( \sum _{i=1}^k {W_i}^{(l)}} \vec {\textrm{x}}_{t+i} \mathrm {+ {b_i}^{(l)}\big )\big ]} \mathrm {+ {b_j}^{(l)} \big \} + {b_m}^{(l)}\big )} \end{aligned}$$

Extracted features $X^{(l)}_t$ is then passed onto the 1D-CNN having hyperbolic tangent ($\Phi _T$) activation function, which is presented in Eq. 3.

3 $$\begin{aligned} \mathrm {X^{(l+1)}_{t}} = \mathrm {\Phi _{T} \big (\sum _{j=1}^k {W_j}^{(l+1)} \big [\Phi _{T} \big ( \sum _{i=1}^k {W_i}^{(l)}} \vec {\textrm{x}}_{t+i} \mathrm { + {b_i}^{(l+1)} \big )\big ]} \mathrm { + {b_j}^{(l+1)} )} \end{aligned}$$

Therefore, it is passed onto the LSTM layer, which has a forget gate ($f_t$), an input gate ($i_t$), a candidate state ($\hat{c}_t$) and an output gate ($o_t$). $\Phi _S$ is the sigmoid activation function. The details are presented in Eqs. 4 to 10.

4 $$\begin{aligned} & \mathrm {f_t = \Phi _{S}\big (W_{1f} {X_t}^{l+1} +W_{2f}{X_{t-1}}^{l+1}+b_f\big )} \end{aligned}$$

5 $$\begin{aligned} & \mathrm {i_t = \Phi _{S}\big (W_{1i}X_t^{l+1} + W_{2i}X_{t-1}^{l+1} + b_i\big )} \end{aligned}$$

6 $$\begin{aligned} & \mathrm {\hat{c_t} = \Phi _{S}\big (W_{1c}X_{t}^{l+1} + W_{2c}X_t^{l+1} + b_c\big )} \end{aligned}$$

Cell-update state ($c_t$),

7 $$\begin{aligned} & \mathrm {c_t = f_t \odot c_{t-1} + i_t \odot \hat{c}_t} \end{aligned}$$

8 $$\begin{aligned} & \mathrm {o_t = \Phi _{S}\big (W_{1o}h_t^{l+1} + W_{2o}h_{t-1}^{l+1} + b_o\big )} \end{aligned}$$

Hidden state ($$h_t$$),

9 $$\begin{aligned} & \mathrm {h_t = o_t \odot \tanh \big ({c_t}\big )} \end{aligned}$$

10 $$\begin{aligned} & \mathrm {H = \sum _{t=1}^{T} h_t} \end{aligned}$$

As this study intends to design a robust multipath classification framework, special interest is laid on feeding raw input signals that are unprocessed with extremely imbalanced target variables into the proposed architecture. DLA is implemented to enhance the model’s ability to capture the spatial and temporal feature dependencies proficiently.

#### Dual limb attention

**Fig. 4:**
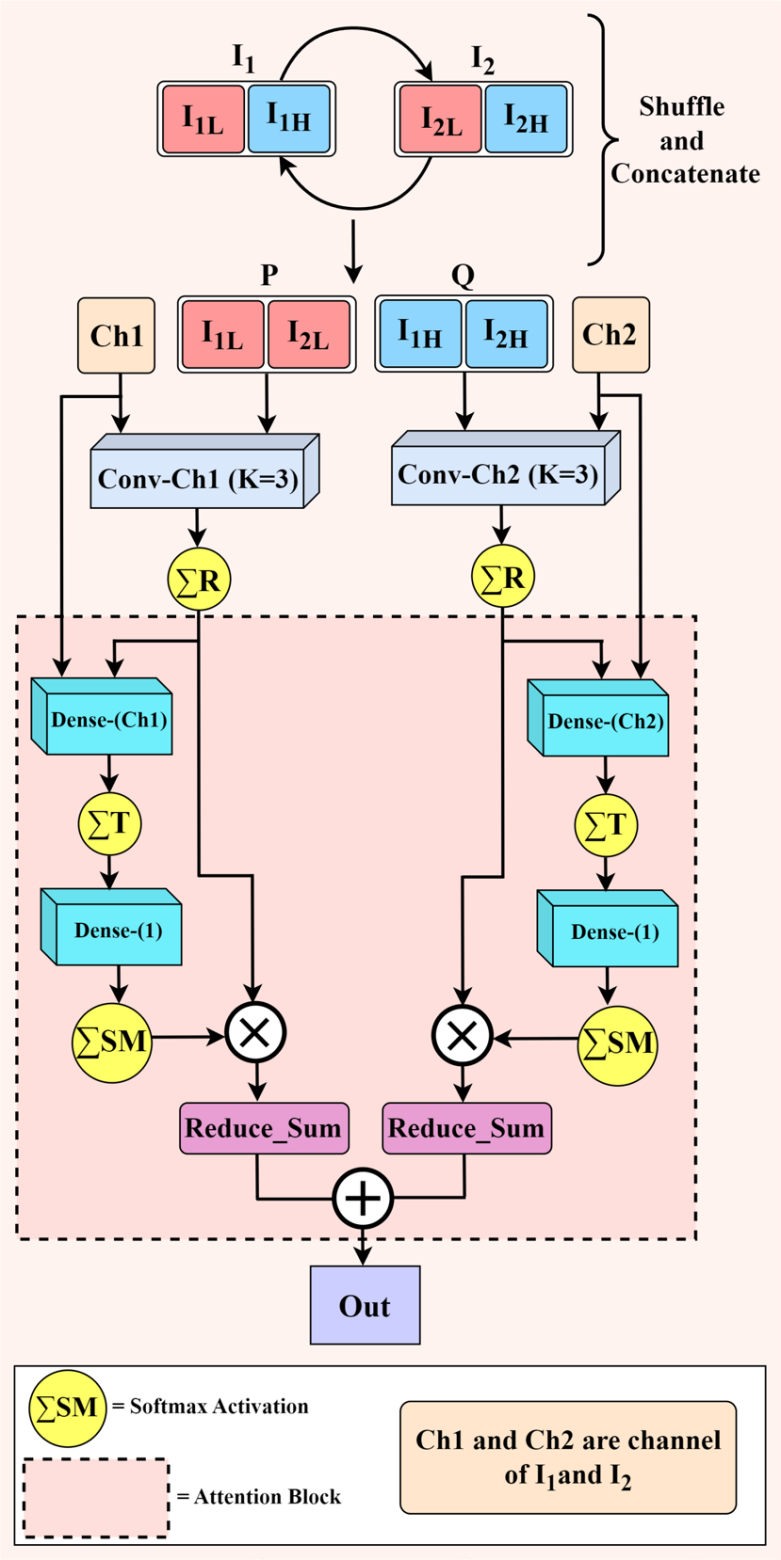
Proposed dual limb attention mechanism module.

To emphasise certain parts of the input, a novel attention mechanism termed as “Dual Limb Attention” is proposed, which is designed specifically for enhancing the feature extraction on the 1D time-series data. Two sets of DLA blocks are introduced to capture the features effectively. The DLA block takes two inputs, which are then split into higher and lower bits by extracting the width and channels of these inputs separately. The similar bits are concatenated. Onto these concatenated blocks, attention is applied to both the higher and lower blocks independently, hence the name “Dual Limb”. Fig. 4 illustrates the structure of the proposed DLA block. The mathematical abstraction of a DLA block is described below.

Let $I_1$ and $I_2$ be the inputs fed into the DLA module, where $I_1$ is the output of the first LSTM layer given by the Eq. 10, and $I_2$ is given by the Eq. 11.

11 $$\begin{aligned} \mathrm {I_2 = \Phi _{R} \big ( \sum _{i=1}^k W_i I_1 +b_i\big )} \end{aligned}$$

Both $I_1$ and $I_2$ are split into higher ($I_{1H}$, $I_{2H}$) and lower bits ($I_{1L}$, $I_{2L}$). The higher and lower bits are concatenated separately as given in Eqs. 12 and 13.

12 $$\begin{aligned} & \mathrm {P = Concat(I_{1L}:I_{2L})} \end{aligned}$$

13 $$\begin{aligned} & \mathrm {Q = Concat(I_{1H}:I_{2H})} \end{aligned}$$

Let *Ch*1 and *Ch*2 be the channel bits of $I_1$ and $I_2$, respectively. *P* and *Q* are fed into two separate CNN layers whose filter size is determined by the length of the channel bits.

14 $$\begin{aligned} & \mathrm {C_1 = \Phi _{R} \big ( \sum _{i=1}^k W_i P + b_i\big )} \end{aligned}$$

15 $$\begin{aligned} & \mathrm {C_2 = \Phi _{R} \big ( \sum _{i=1}^k W_i Q +b_i\big )} \end{aligned}$$

Applying attention on $C_1$ and $C_2$ separately,

16 $$\begin{aligned} & \mathrm {C_3 = \Phi _{SM} \big \{\big [\Phi _{T}\big (W_{D_{ch1}}C_1 + b_{D_{ch1}}\big )\big ] W_{D_1} + b_{D_1}\big \} } \end{aligned}$$

17 $$\begin{aligned} & \mathrm {C_4 = \Phi _{SM} \big \{\big [\Phi _{T}\big (W_{D_{ch2}}C_2 + b_{D_{ch2}}\big )\big ] W_{D_1} + b_{D_1}\big \} } \end{aligned}$$

$\Phi _{SM}$ = Softmax Activation Function

18 $$\begin{aligned} & \mathrm {L_1 = reduce\_sum\big (C_3 \times C_1\big )} \end{aligned}$$

19 $$\begin{aligned} & \mathrm {L_2 = reduce\_sum\big (C_4 \times C_2\big )} \end{aligned}$$

20 $$\begin{aligned} & \mathrm {O_{DLA} = L_1 + L_2} \end{aligned}$$

After being processed by two DLA blocks, the features are passed on to the LSTM layer, followed by CNN and fully connected dense layers, to finally arrive at the output node $\hat{y}$, which bears two distinct classes, distinguished using the sigmoid activation function. In Eq. 21, z is the intermediate output from the final dense layer.

21 $$\begin{aligned} \mathrm {\hat{y} = {\left\{ \begin{array}{ll}1, & \Phi _{S}\big (z\big ) \ge 0.5 \\ 0, & \Phi _{S}\big (z\big ) < 0.5 \end{array}\right. } } \end{aligned}$$

To nullify the effect of extreme class imbalance associated with the target classes, a novel loss strategy termed as “Alternating Loss Strategy (ALS)” is proposed and detailed in “Alternating loss strategy” section. The pseudo code of MOVENetx64 is presented in Algorithm 1.

**Algorithm 1:**
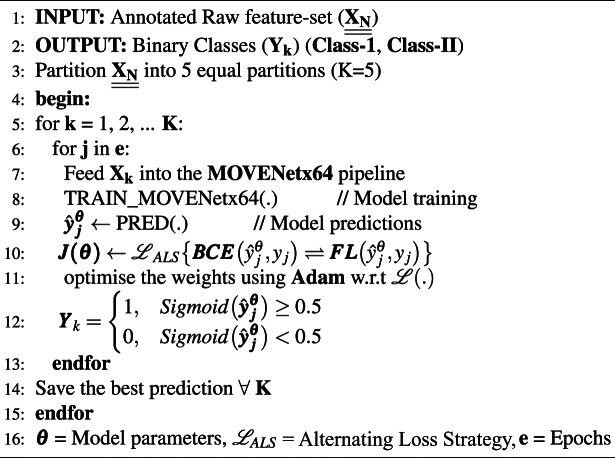
MOVENetx64 with DLA and ALS.

## Training and implementation

### Dataset

Three high-quality datasets representing city, suburban, and highway vehicular scenarios are generated using 3D ray-tracing from the Remcom Wireless InSite simulator-DeepMIMO^48^. The network attributes for each use-case are reported in Table 2, which are fed into the DeepMIMO generator. User density is set according to the annual report of the Telecom Regulatory Authority of India (TRAI) 2023–24^49^ to emulate near-real-time conditions.

**Table 2:** Network attributes of the dataset.

Data attributes	Values		
System bandwidth	500 MHz		
Driving scenario	City	Suburban	Highway
Dynamic scenes	NA	NA	Scene 1-10
Basestations	1	5	2
Users	965090	253400	88670
Size of antenna	x=1; y=32; z=8		
Antenna spacing	0.5$\lambda$		
Active paths	15	15	10

### Data preparation

The data generated using the DeepMIMO platform consists of the channel matrix and the channel impulse response associated with the base-stations and their respective user links. The prominent features, such as the ToA, Zenith and Azimuth angle of arrival and departure, phase, and the received power associated with each of the user links, are extracted to form the input vector ${\bf X}$, which is mathematically given by Eq. (22) for **“m”** MPCs.

22 $$\begin{aligned} \begin{aligned} \vec {\textrm{X}} = \mathrm {f \big (\alpha _{m}, \tau _{m}, \Theta _{R,m}, \Theta _{T,m}, \varphi _{R,m}, \varphi _{T,m}\big )} \end{aligned} \end{aligned}$$

Similarly, the multipath status of each BS-MU link is extracted and annotated to compose the target output class. To rule out the bias associated with multi-class classification problems, this study employs a unique class mapping, which combines the LOS and FOMP links as Class-I and the HOMPs are grouped to Class-II. Hence, the primary focus lies in distinguishing Class-II to mitigate the presence of HOMPs efficiently.

**Fig. 5:**
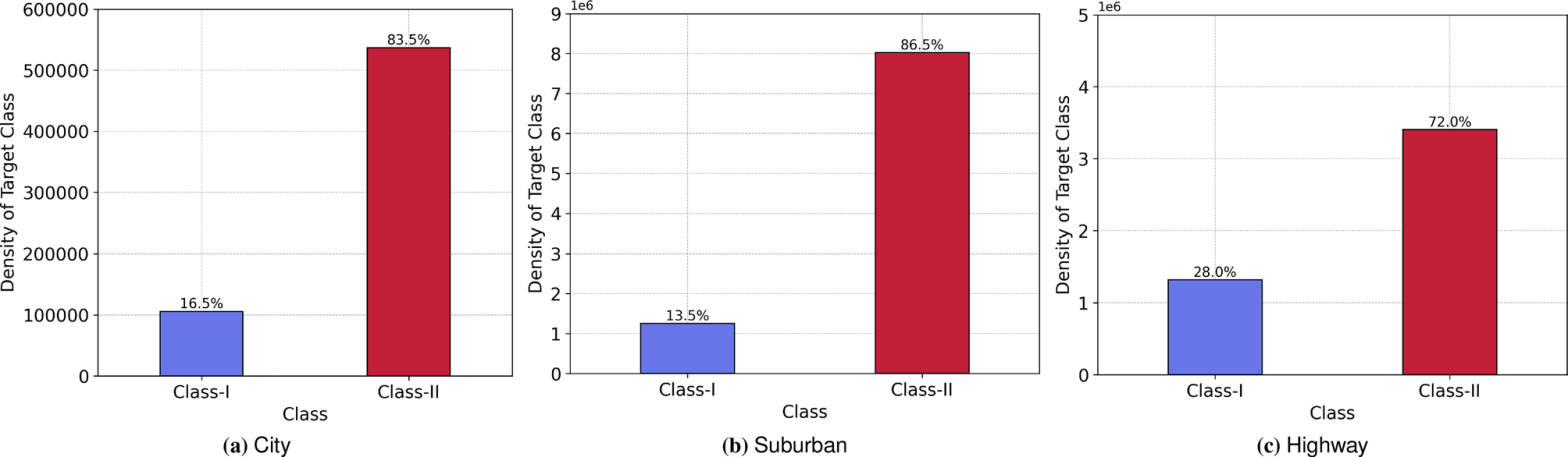
Density plot of target class.

**Table 3:** Distribution of dataset samples.

Vehicular scenario	Samples		
	Train	Validate	Test
City	449801	96386	96387
Suburban	6493071	1391372	1391373
Highway	3305964	708421	708421

Due to the potential obstacles present in the vehicular networks, the density of HOMPs is higher than that of the direct links and FOMPs, which is depicted in Fig. 5 for all the diverse use-cases considered. ALS, a specialised loss mechanism, is developed to mitigate the effect of an imbalanced target class, as detailed in “Alternating loss strategy” section. The distribution of data samples for all driving scenarios used for training, validation, and testing the proposed MOVENetx64 is detailed in the Table 3.

### Performance metrics

A comprehensive set of performance metrics is employed to evaluate the effectiveness of the proposed MOVENetx64 model. Alongside the traditional metrics like accuracy, precision, recall, F1-score and Area Under a Receiver Operator Curve (AUC), two prominent metrics-Cohen Kappa (CK) and HOMP Prediction error (HOMPPE) are considered to handle extreme class imbalance^50^. CK is best suited for imbalanced classification as it considers the chance agreement. On the contrary, accuracy can mislead the model when one class dominates over the other. Thereby, CK highlights the model’s true predictive potential.

23 $$\begin{aligned} & {\bf HOMPPE} = \mathrm {\frac{FP}{FP + TN} } \end{aligned}$$

24 $$\begin{aligned} & \mathbf {Cohen-Kappa} = \mathrm {\frac{ \big ( P_{o} - P_{e} \big ) }{ \big (1 - P_{e} \big ) }} \end{aligned}$$

25 $$\begin{aligned} & \mathbf {P_{o}} = \mathrm {\frac{TP + TN}{TP + FP + TN + FN}} \end{aligned}$$

26 $$\begin{aligned} & \mathbf {P_{e}} = \mathrm {\frac{\big (TP + FN\big ) \big (TP + FP\big ) \big (FP + TN\big ) \big (FN + TN\big )}{ \big ( TP + FP + TN + FN \big )^{2}}} \end{aligned}$$

where, $\mathbf {P_o}$ is the observed agreement; $\mathbf {P_e}$ is the expected agreement

As this study is based on classifying the different multipaths, a dedicated metric HOMPPE^47^ is utilised to analyse the model’s ability to eliminate HOMP with minimal prediction error. Eqs. 23–26 provide the mathematical interpretation of the performance metrics in terms of true positive (TP), false positive (FP), true negative (TN), and false negative (FN).

### Alternating loss strategy (ALS)

To address the class imbalance observed in “Dataset” section, two prominent loss functions-Binary Cross Entropy (BCE) and Focal Loss (FL) are employed. The proposed model was trained on BCE until the loss became stagnant. If no improvement was observed after 10 epochs, the training was switched to FL. This process was repeated for the entire training stage.

27 $$\begin{aligned} & {\bf BCE} = \mathrm { - \frac{1}{N} \sum _{i=1}^N [ y_{i} \log {\big (\hat{y_{i}}\big )} + \big (1 - y_{i} \big ) \log { \big (1 -\hat{y_{i}}\big )}]} \end{aligned}$$

28 $$\begin{aligned} & {\bf FL} = \mathrm {- \alpha {\big (1- \hat{y_i}} \big )^{ \gamma } \log \hat{y}} \end{aligned}$$

where, $y_i$ (True label) and ${\hat{y}}_i$ (Predicted probability) of the ith data sample; *N* is the total number of data samples $\alpha$ = 0.25, $\gamma$ = 2.0

As BCE is sensitive to class imbalance, it struggled to improve the F1-score, whereas FL effortlessly uplifted F1-scores, but failed to improve the prediction accuracy. Hence, a novel loss-alternating mechanism was designed to leverage the strengths of both loss functions, while addressing their limitations through the appropriate selection of weights $\alpha$ and $\gamma$, as represented in Eqs. 27 and 28. The workflow of ALS is described using a pseudo-code presented in Algorithm 2.

**Algorithm 2:**
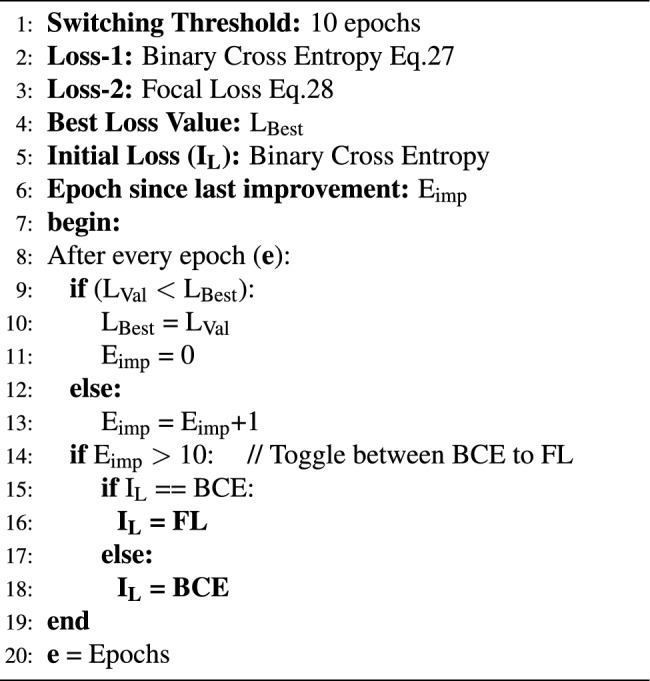
Alternating loss strategy.

### Experimental setup

The experiments are conducted using DL library Keras with TensorFlow as the backend on a 40 Core 2x Xeon G-6248 processor with 2x NVIDIA V100 cards. The proposed model is trained using the Adaptive Moment (ADAM) optimiser in a batch size of 1000 samples for 500 epochs. A learning rate scheduler based on ReduceLRonPlateau is employed to enable fine-tuning of the proposed model. To overcome any chances of over-fitting, a 5-fold cross-validation is employed, along with early stopping and an L2 regulariser. Table 4 summarises the hyper-parameters of the proposed model.

**Table 4:** The hyper-parameters of the proposed model.

Parameters	Value	
Optimiser	ADAM	
Learning rate	ReduceLROnPlateau	
	Patience	5
	Min learning rate	0.0001
Dropout	0.05	
Epochs	500	
Batch size	1000	
K-fold	05	
Loss	Binary cross-entropy	
	Focal loss	

**Fig. 6:**
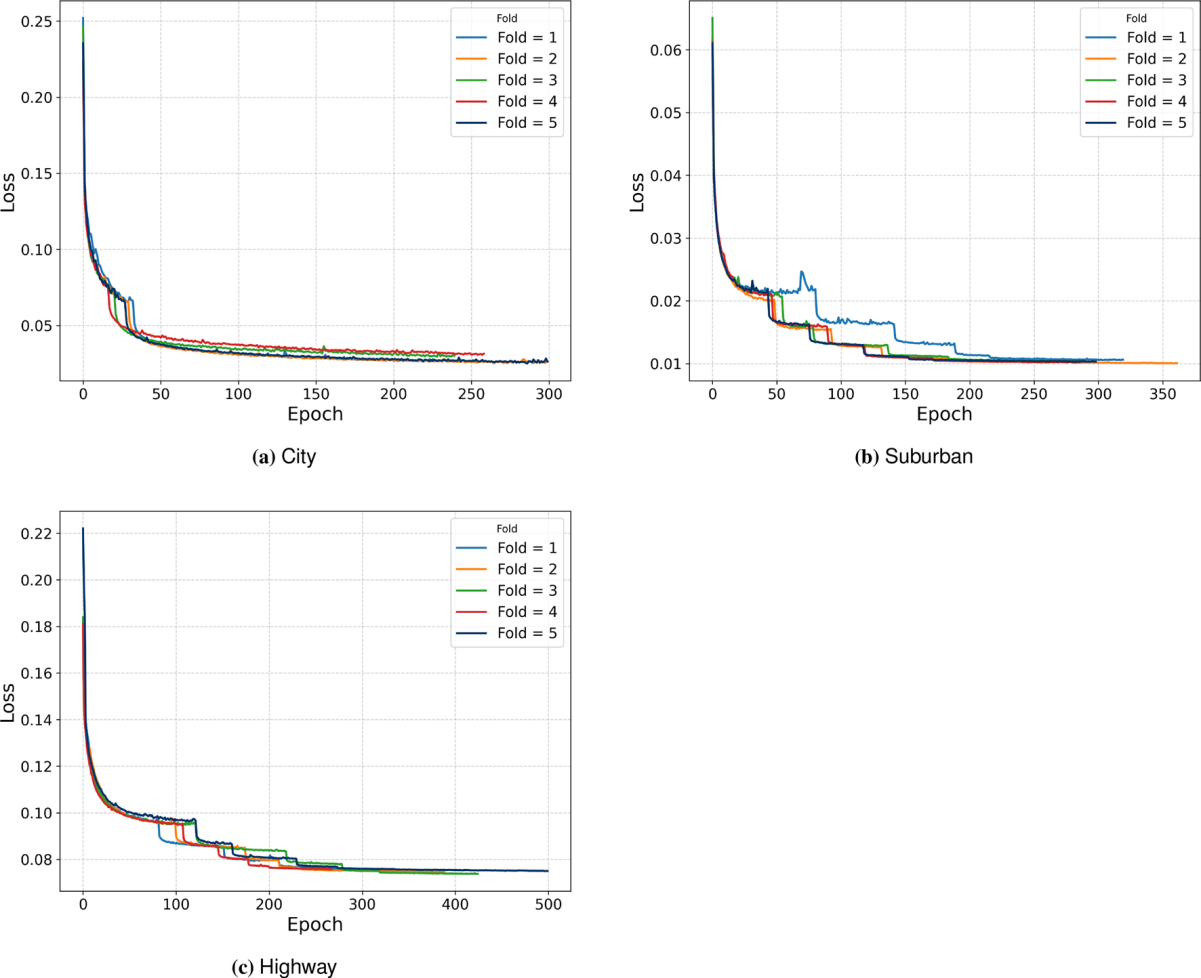
Learning curves for loss.

Fig. 6 illustrates the validation loss curves obtained for all five-fold cross-validation conducted across City, Suburban and Highway scenarios. The smooth convergence of the validation curves justifies the optimal training, thereby dismissing any chance of the proposed model being overfit or underfit.

## Results and discussion

This section presents the ablation study, experimental outcome of the proposed MOVENetx64 model along with a comparative analysis between the standard DL-based classifiers such as Deep Neural Network (DNN)^51^, LeNet-5^52^, AlexNet^53^, VGG-16^54^, ResNet-32^55^, ResNet-64^55^, LSTM^51^ and BiLSTM^51^. Also, the proposed model is compared with the state-of-the-art (SOTA) multipath classification models recorded in the literature.

### Ablation study of the proposed model

The proposed MOVENetx64 is designed and developed by experimentally verifying the contributions of individual architectural components, such as LSTM, Dual Limb Attention, filter size, and the role of an additional CNN layer in DLA. Five intermediate models (IM) are developed to analyse their ability to detect multipaths effectively. Table 5 represents the architectural layers of each IM.

**Table 5:** Intermediate models of the proposed MOVENetx64.

Model	Layer				
	LSTM	DLA	Attention	Filter	Additional CNN
IM1	✓	✗	✗	64	✓
IM2	✓	✓	✗	64	✓
IM3	✗	✓	✓	64	✓
IM4	✓	✓	✓	32	✓
IM5	✓	✓	✓	64	✗
**MOVENetx64(Proposed)**	**✓**	**✓**	**✓**	**64**	✓

Significant values are in bold.

All these models are trained and evaluated on the proposed alternating loss strategy. The primary focus of this study is to develop a model that minimises the HOMP prediction error, thereby validating the precise identification and classification of HOMPs. The experimental outcome of each of these models is presented in Table 6.

#### Intermediate Model 1

In IM1, the dual limb attention mechanism is omitted, while the LSTM and convolutional neural network layers are retained, with a uniform filter size of 64 throughout the model. This model achieves an accuracy of 99.36%, an F1-score of 97.63%, an AUC of 99.997%, a Cohen-Kappa of 97.25% and a HOMP prediction error of 0.0042.

#### Intermediate Model 2

In IM2, a DLA layer without the attention mechanism is introduced, alongside LSTM, maintaining the same filter size. An accuracy of 99.37%, F1-score of 97.67%, AUC of 99.998%, Cohen-Kappa of 97.31% and HOMP prediction error of 0.33% is rendered by IM2. With the addition of the DLA layer, there has been a decrease of 21.42% in the HOMPPE value compared to IM1, demonstrating the effectiveness of the DLA layer.

#### Intermediate Model 3

The DLA layer with attention is introduced, omitting LSTM layers, to form IM3. This model achieves an accuracy of 99.40%, an F1-score of 97.75%, an AUC of 99.998%, a Cohen-Kappa of 97.40% and a HOMP prediction error of 0.0029%. The introduction of an attention mechanism within the DLA has enhanced the ability of DLA by further reducing the HOMPPE value of IM2 by 12.12%.

#### Intermediate Model 4

To analyse the importance of the filter size, IM4 is developed with a filter size of 32 while retaining the architectural components of the proposed MOVENetx64. With IM4, the HOMP prediction error increased by 52.94% compared to the proposed MOVENetx64 architecture.

#### Intermediate Model 5

The role of providing distinct inputs to DLA is analysed by overriding the additional CNN layer, resulting in identical inputs being fed to the attention mechanism. Although IM5 has exhibited a satisfactory performance across the classification metrics, the HOMP prediction error has increased to 88.23% compared to the proposed model. This confirms the role of an additional CNN layer, which fine-tunes the features further, thereby enhancing the overall performance.

The proposed model delivers an effective performance compared to all the intermediate models, which justifies the significance of each architectural component.

**Table 6:** Ablation study of the proposed MOVENetx64.

Model	Metrics						
	Precision	Recall	Accuracy	F1-score	AUC	HOMPPE	Cohen-Kappa
IM1	0.9735	0.9792	0.9936	0.9763	0.9997	0.0042	0.9725
IM2	0.9791	0.9745	0.9937	0.9767	0.9998	0.0033	0.9731
IM3	0.9810	0.9741	0.9940	0.9775	0.9998	0.0029	0.9740
IM4	0.9829	0.9672	0.9933	0.9748	0.9997	0.0026	0.9710
IM5	0.9788	0.9691	0.9929	0.9610	0.9993	0.0032	0.9673
**MOVENetx64(Proposed)**	**0.9887**	**0.9670**	**0.9941**	**0.9638**	**0.9998**	**0.0017**	**0.9750**

Significant values are in bold.

### Ablation study of the feature set

As this study considers a combination of time, power, angle and phase characteristics associated with the multipaths, an ablation study is conducted to study the importance of each feature set in improving the overall performance of the proposed MOVENetx64. Six input feature combinations are fed into the proposed MOVENetx64, and the performance rendered by each of them is recorded in Table 7.

**Table 7:** Ablation study of the input features.

Feature combination	Features				Metrics			
	Angle characteristics	Received power	Time of arrival	Phase	Accuracy	F1-score	AUC	HOMPPE
F1	✓	✗	✗	✗	0.9928	0.9606	0.9996	0.0036
F2	✗	✓	✗	✗	0.8919	0.7903	0.8679	0.0113
F3	✗	✗	✓	✗	0.8709	0.1357	0.8475	0.0081
F4	✗	✗	✗	✓	0.8649	0.0	0.0	Undefined
F5	✓	✓	✓	✗	0.9933	0.9620	0.9994	0.0043
**F6 (Proposed)**	✓	✓	✓	✓	**0.9941**	**0.9638**	**0.9998**	**0.0017**

Significant values are in bold.

To assess the contribution of individual features, F1, F2, F3, and F4, corresponding to angle characteristics, received power, ToA, and phase, respectively, are formed. An improved performance is achieved on F1 compared to F2, F3, and F4, validating that angle characteristics are the most important feature, followed by received power, ToA, and phase, as reported in F2, F3, and F4, respectively. As F4 demonstrates the least performance, the F5 feature combination is formed by omitting the phase information. It is to be noted that, though the phase information alone could not deliver promising performance, it helps fine-tune overall performance as reported by the proposed feature combination F6.

Hence, confirming that the angle characteristics and received power are the necessary features to identify the HOMPs effectively, whereas ToA and phase help in fine-tuning the feature-set. F6 improves classification accuracy and HOMP prediction error by 0.1309% and 52.77%, respectively, compared to F1. In contrast to F5, an improvement of 0.0805% and 60.46% is achieved with F6 on the classification accuracy and HOMP prediction error.

### Comparative analysis

The proposed MOVENetx64 model is compared with standard DL classifiers and the benchmark multipath classifiers recorded in the literature.

#### Comparison with standard DL-classifiers

The performance of the proposed MOVENetx64 and the standard DL classifiers is comprehensively evaluated on three diverse datasets described in “Dwataset” section. A detailed experimental analysis of each model is presented in Table 8. The results are recorded from the five-fold cross-validation conducted on three driving scenarios. From a primitive DNN model to sophisticated ResNet models, this study has conducted an exhaustive evaluation of the performance of prime classifiers to validate the effectiveness of the proposed model. To evaluate the performance improvement of the proposed model, the best result delivered by the DL-classifiers is chosen for comparison.

**Table 8:** Comparison of performance metrics of standard deep learning classifiers.

Dataset	Metrics	Precision	Recall	F1-score	Accuracy	AUC	HOMPPE	Cohen-Kappa
	Model							
City	DNN	0.8486	0.7784	0.8201	0.9416	0.8383	0.0307	0.7853
	LeNet	0.8686	0.7705	0.8163	0.9418	0.8567	0.0259	0.7818
	AlexNet	0.9013	0.8616	0.8841	0.9358	0.9093	0.0332	0.8381
	VGG-16	0.9030	0.8776	0.8919	0.9405	0.9030	0.0368	0.8515
	ResNet-32	0.9031	0.8743	0.8800	0.9342	0.9013	0.0365	0.8345
	ResNet-64	0.9057	0.8850	0.8765	0.9395	0.8975	0.0392	0.8494
	LSTM	0.8532	0.7766	0.7992	0.9394	0.8886	0.0180	0.7640
	BiLSTM	0.8558	0.7845	0.8161	0.9419	0.8589	0.0253	0.7817
	**MOVENetx64(Proposed)**	**0.9692**	**0.9520**	**0.9539**	**0.9871**	**0.9982**	**0.0060**	**0.9494**
Suburban	DNN	0.9714	0.8884	0.9278	0.9810	0.9586	0.0060	0.9169
	LeNet	0.9701	0.8763	0.9203	0.9796	0.9797	0.0028	0.9087
	AlexNet	0.9670	0.8899	0.9314	0.9822	0.9777	0.0031	0.9213
	VGG-16	0.9691	0.9024	0.9358	0.9837	0.9738	0.0038	0.9287
	ResNet-32	0.9649	0.9161	0.8484	0.9802	0.9484	0.0077	0.9143
	ResNet-64	0.9658	0.9245	0.9172	0.9836	0.9663	0.0049	0.9286
	LSTM	0.9750	0.8532	0.9086	0.9769	0.9780	0.0029	0.8955
	BiLSTM	0.9708	0.8733	0.9207	0.9798	0.9816	0.0025	0.9092
	**MOVENetx64(Proposed)**	**0.9887**	**0.9670**	**0.9638**	**0.9941**	**0.9998**	**0.0017**	**0.9750**
Highway	DNN	0.9042	0.8544	0.8776	0.9336	0.9060	0.0342	0.8321
	LeNet	0.9024	0.8466	0.8779	0.9336	0.9045	0.0349	0.8323
	AlexNet	0.9026	0.8666	0.8895	0.9395	0.9095	0.0336	0.8479
	VGG-16	0.9046	0.8724	0.8837	0.9393	0.9046	0.0357	0.8477
	ResNet-32	0.9098	0.8848	0.8827	0.9377	0.8818	0.0465	0.8460
	ResNet-64	0.9078	0.8856	0.8763	0.9387	0.9077	0.0343	0.8459
	LSTM	0.9044	0.8472	0.8644	0.9292	0.9286	0.0240	0.8169
	BiLSTM	0.9030	0.8479	0.8717	0.9312	0.9084	0.0326	0.8249
	**MOVENetx64(Proposed)**	**0.9405**	**0.9312**	**0.9321**	**0.9644**	**0.9957**	**0.0228**	**0.9118**

Significant values are in bold.

**Fig. 7:**
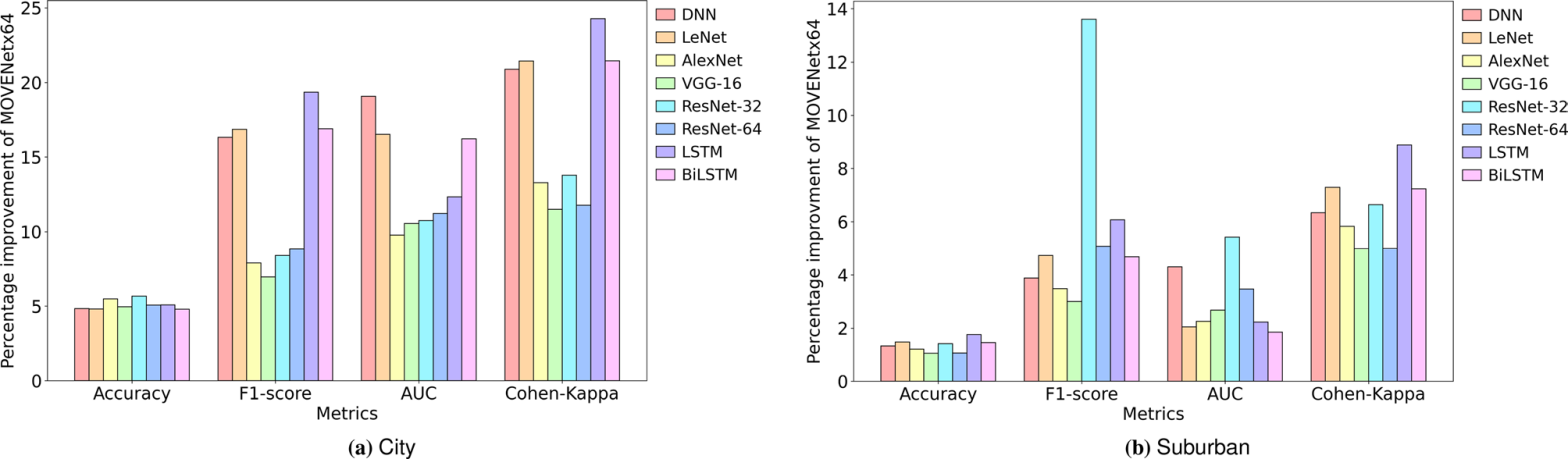
Performance improvement of MOVENetx64 against the DL classifiers on city and suburban use-cases.

Under the City driving condition, the proposed MOVENetx64 model outperforms the standard DL classifiers across all performance metrics, yielding a Precision of 96.92%, a Recall of 95.20%, an F1-score of 95.39%, an Accuracy of 98.71%, an AUC of 99.82%, an HOMPPE of 0.0060, and a Cohen Kappa of 94.94%. MOVENetx64 has outperformed standard DL classifiers by achieving an improvement of 7.01% (ResNet-64), 7.57% (ResNet-64), 6.95% (VGG-16), 4.79% (BiLSTM), 9.77% (AlexNet), 11.49% (LSTM) and 66.66% (VGG-16) on the values of precision, recall, F1-score, Accuracy, AUC, Cohen-kappa and HOMPPE metrics, respectively. The percentage improvement showcased by MOVENetx64 over all these models for the city use-case is depicted in Fig. 7a.

For the Suburban driving scenario, MOVENetx64 has exhibited an exceptional performance by achieving a Precision of 98.87%, Recall of 96.70%, F1-score of 96.38%, Accuracy of 99.41%, AUC of 99.98%, HOMPPE of 0.0017 and Cohen-kappa of 97.50%. Compared to the standard DL classifiers, MOVENetx64 showcased a percentage improvement of 1.40% (LSTM), 4.59% (ResNet-64), 2.99% (VGG-16), 1.05% (VGG-16), 1.85% (BiLSTM), 4.98% (VGG-16) and 32% (BiLSTM) on the values of precision, recall, F1-score, Accuracy, AUC, Cohen-kappa and HOMPPE metrics, respectively. Fig. 7b presents the percentage improvement of MOVENetx64 against other DL classifiers for the suburban driving scenario.

**Fig. 8:**
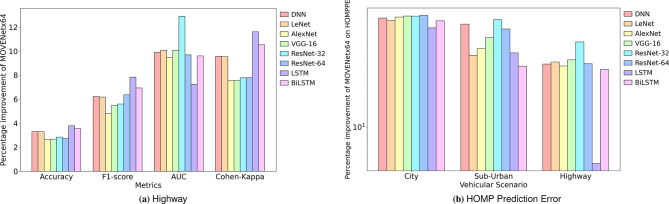
Performance improvement of MOVENetx64 against the DL classifiers.

Similarly, MOVENetx64 has demonstrated a promising competence on the Highway scenario by delivering a Precision of 94.05%, Recall of 93.12%, F1-score of 93.21%, Accuracy of 96.44%, AUC of 99.57%, HOMPPE of 0.0228 and Cohen-kappa of 91.18%. The proposed model achieved a percentage improvement of 3.37% (ResNet-32), 5.14% (ResNet-64), 4.78% (AlexNet), 2.65% (AlexNet), 7.22% (LSTM), 7.53% (AlexNet) and 5% (LSTM) on the values of precision, recall, F1-score, Accuracy, AUC, Cohen-kappa and HOMPPE metrics, respectively. Fig. 8a presents the percentage improvement of MOVENetx64 against other DL classifiers for the highway driving scenario.

Exceptional values of F1-score and AUC highlight the balanced sensitivity-specificity trade-off. Substantially higher Cohen Kappa values reflect a strong agreement between the predicted class and ground truth beyond random chance. As this study aims to distinguish the HOMPs effectively, a keen interest is laid to analyse the HOMP prediction error delivered. Fig. 8b presents the percentage improvement achieved by MOVENetx64 on the HOMPPE over the DL classifiers. The proposed model has exhibited 84.69%, 77.92%, and 50.96% improvements in HOMP prediction error on City, Suburban, and Highway scenarios, respectively. This improved performance is attributed to the efficient feature extraction performed by the DLA block, which delivers promising results even on raw, unprocessed data.

**Fig. 9:**
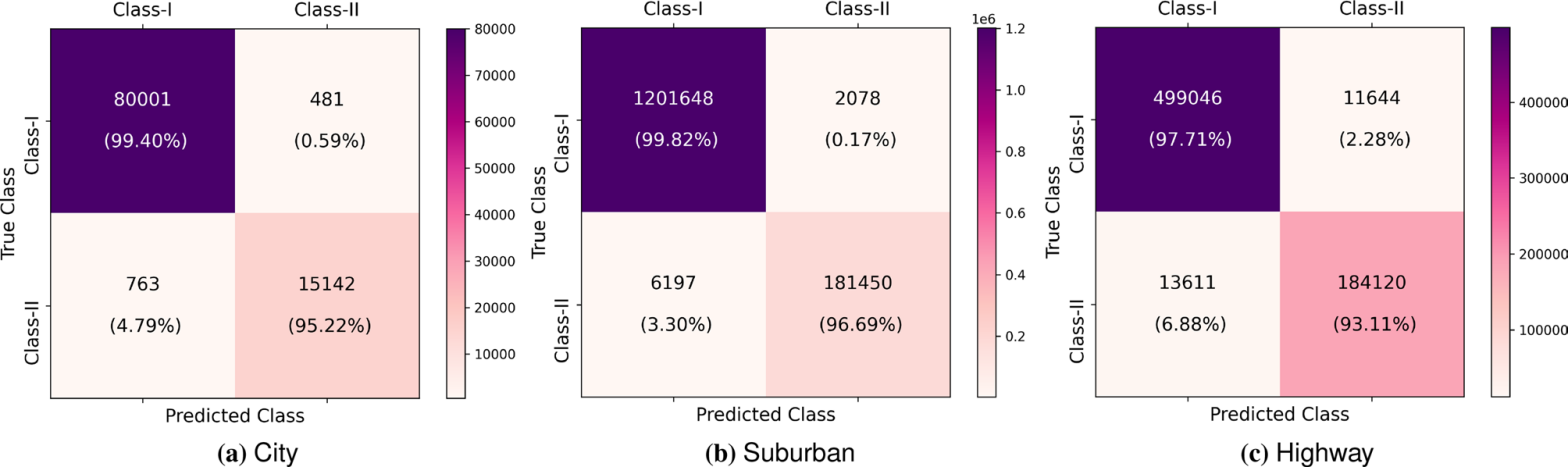
Confusion matrices of the proposed MOVENetx64.

A confusion matrix is of great help in evaluating the effectiveness of the DL models, as it provides detailed information on the number of samples that are classified correctly and falsely. Fig. 9 presents the confusion matrices of MOVENetx64 when evaluated on City, Suburban, and Highway driving use cases. It is worth noting that MOVENetx64 has been efficient in maintaining the lowest possible misclassification rates of 1.29%, 0.59%, and 3.56% in city, suburban, and highway environments, respectively. The low misclassification rate justifies the promising nature of the proposed model in accurately identifying the type of multipaths in diverse use cases.

#### Comparison with the benchmark multipath classification models

**Fig. 10:**
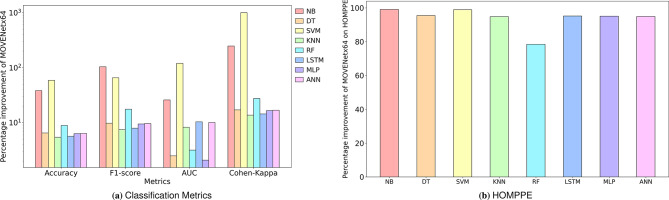
Performance improvement of MOVENetx64 against the benchmark multipath classifiers.

The proposed MOVENetx64 model is bench-marked against SOTA models such as Naive-Bayes (NB)^47^, Decision Tree (DT)^47^, Support Vector Machine (SVM)^11,47^, K-Nearest Neighbours (KNN)^11,47^, Random Forest (RF)^47^, LSTM^11^, MLP^11^ and ANN^11^ reported in the literature. The experimental outcomes of each of these models, alongside MOVENetx64, are reported in the Table 9. The proposed model demonstrates an improved performance over the state-of-the-art models by a margin of 5.40% (KNN), 1.73% (RF), 9.64% (KNN), 7.45% (KNN), 2.08% (MLP), 13.67% (KNN) and 78.48%(RF) on the values of Accuracy, Precision, Recall, F1-score, AUC, Cohen-kappa and HOMP prediction error. Fig. 10a, and Fig. 10b present the percentage improvement achieved by MOVENetx64 against the SOTA models on the classification metrics and HOMP prediction error, respectively.

**Table 9:** Comparative analysis of benchmark multipath classifiers.

Metrics							
Model	Accuracy	Precision	Recall	F1-Score	AUC	CK	HOMPPE
NB^47^	0.7189	0.4956	0.4504	0.4719	0.7937	0.2810	0.1772
DT^47^	0.9336	0.8980	0.8596	0.8784	0.9755	0.8328	0.0377
SVM^11,47^	0.6258	0.5402	0.6305	0.5818	0.4543	0.1086	0.1555
KNN^11,47^	0.9431	0.9124	0.8819	0.8969	0.9240	0.8577	0.0329
RF^47^	0.9131	0.9718	0.7090	0.8198	0.9691	0.7644	0.0079
LSTM^11^	0.9410	0.9137	0.8730	0.8930	0.9061	0.8523	0.0353
MLP^11^	0.9352	0.9052	0.8578	0.8807	0.9794	0.8363	0.0348
ANN^47^	0.9347	0.9241	0.8218	0.8794	0.9083	0.8347	0.0333
**MOVENetx64(Proposed)**	**0.9941**	**0.9887**	**0.9670**	**0.9638**	**0.9998**	**0.9750**	**0.0017**

Significant values are in bold.

The proposed model consistently outperforms all the standard DL classifiers and the state-of-the-art models across every evaluation metric. MOVENetx64 achieves the highest Precision, Recall, and F1-score, indicating superior ability rendered by Dual Limb attention to correctly identify positive cases while maintaining a balance between false positives and false negatives. The Accuracy further confirms its reliability in overall classification, while the AUC underscores its robustness in distinguishing between classes across decision thresholds. Finally, the Cohen’s Kappa score, which accounts for chance agreement, confirms that the model exhibits higher predictive power and more reliable agreement with the true labels.

Collectively, these results demonstrate that the proposed MOVENetx64 model is not only the top performer on individual metrics but also the most stable and generalisable solution, and overall establishes it as the best choice for distinguishing the type of multipaths.

### Complexity analysis

A well-performing DL model should also be characterised with minimal model complexity and processing time. The complexity analysis comparing the proposed model with the standard DL classifiers is presented in the Table 10. Despite being developed with minimal parameters, MOVENetx64 outperformed the standard DL classifiers. A lower computational time than deeper architectures, such as Residual-Net, whose performance was significantly inferior to MOVENetx64, indicates the balance between a lightweight design and the high predictive potential of MOVENetx64. Hence, MOVENetx64 can be well-suited for deployment in resource-constrained environments, such as ITS.

The complexity analysis of benchmark SOTA models is presented in the Table 11. Though MOVENetx64 takes a longer prediction time than the SOTA models, it renders a higher margin of performance improvement over the SOTA models on multiple driving scenarios. Hence, MOVENetx64, with its dual attention mechanism, can effectively distinguish between multipaths and thereby mitigate the effect of HOMPs.

**Table 10:** Complexity analysis of standard DL classifiers.

Model	Parameters M=10$^{6}$	Time (s)		Loss
		Train	Test	
DNN	9345	32	40	0.0605
LeNet	12821	34	55	0.0619
LSTM	16961	69	48	0.0708
BiLSTM	33921	107	51	0.0638
ResNet-34	7.24M	249	292	0.0515
ResNet-64	14.74M	548	500	0.0404
VGG-16	18.41M	110	78	0.0432
AlexNet	18.94M	106	67	0.0505
**MOVENetx64(Proposed)**	**223429**	**198**	**88**	**0.0100**

Significant values are in bold.

**Table 11:** Comparative analysis of benchmark multipath classifiers.

Model	Parameters	Time (s)	
		Train	Test
NB	NA	0.8087	0.0806
DT	NA	24.1384	0.0684
KNN	NA	11.2628	27.1488
SVM	NA	15983.4524	2601.4946
RF	NA	64.0890	0.8392
MLP	1021	460.6150	20.6087
LSTM	452551	28771.2968	43.4742
ANN	3,641	700.5773	20.6070
**MOVENetx64(Proposed)**	**223429**	**198**	**88**

Significant values are in bold.

The exhaustive experimental evaluation of the proposed MOVENetx64, in comparison to the standard DL classifiers and the benchmark multipath classifiers, concludes that MOVENetx64 exhibits superior performance among all the models considered. The presence of a specialised DLA mechanism has enhanced feature extraction, resulting in promising performance across diverse use cases. It is worth noting that the standard DL classifiers outperformed the benchmark multipath classifiers, which validates the importance of DL models over basic ML approaches. Hence, the study is extended to evaluate the performance of the proposed MOVENetx64 and the standard DL classifiers on the DeepSense6G^56^-a publicly available dataset.

### Experimental evaluation on the DeepSense6G data

The DeepSense6G^56^ dataset is utilised to analyse the performance of the proposed MOVENetx64 and DL classifiers. This data comprises of the received power alone as the key input feature, along with the associated target class label. Table 12 presents the performance metrics delivered by the multipath classifiers on DeepSense6G data. MOVENetx64 achieved a classification accuracy of 94.02% and a HOMP prediction error of 6.87%, thereby outperforming the DL classifiers by 6.59% (VGG-16) and 54.71% (VGG-16) in terms of accuracy and HOMPPE. The absence of critical channel features associated with time and angular characteristics, along with limited data samples, has resulted in a dip in the overall performance reported.

**Table 12:** Experimental outcome of the multipath classifiers on the DeepSense6G dataset.

Metrics							
Model	Accuracy	Precision	Recall	F1-score	AUC	CK	HOMPPE
DNN	0.8025	0.8018	0.8036	0.8027	0.8926	0.6049	0.1987
LeNet	0.8393	0.8167	0.8750	0.8448	0.9380	0.6786	0.1964
LSTM	0.7165	0.7156	0.7188	0.7171	0.7228	0.4330	0.2857
BiLSTM	0.7210	0.7181	0.7277	0.7228	0.8167	0.4420	0.2857
ResNet-34	0.5536	0.5321	0.8884	0.6656	0.5882	0.1071	0.7812
ResNet-64	0.5871	0.5552	0.8750	0.6794	0.6421	0.1741	0.7009
VGG-16	0.8820	0.8564	0.9180	0.8849	0.9635	0.7631	0.1517
AlexNet	0.8516	0.8208	0.8996	0.8584	0.9615	0.7031	0.1964
MLP	0.7701	0.7854	0.7433	0.7638	0.8622	0.5402	0.2031
LSTM	0.7891	0.7884	0.7902	0.7893	0.8625	0.5781	0.7893
ANN	0.7902	0.7941	0.7835	0.7888	0.8826	0.5804	0.2031
**MOVENetx64** **(Proposed)**	**0.9402**	**0.9354**	**0.9456**	**0.9399**	**0.9666**	**0.8766**	**0.0687**

Significant values are in bold.

## Conclusion

This work demonstrated the efficiency of the proposed novel deep learning architecture, MOVENetx64, in reliably detecting the high-order multipaths in a vehicular communication system. Traditional deterministic techniques are penalised by their high complexity and low robustness in differentiating first-order and higher-order multipath components. The proposed architecture was developed using convolutional neural networks, Long Short-Term Memory layers, and a novel dual limb attention mechanism to effectively extract temporal and spatial features from unprocessed raw data. Further, a novel alternating loss strategy was employed to override the extreme class imbalance associated with the target classes. Three diverse vehicular scenarios, including City, Suburban, and Highway, were generated using the ray tracing technique. A combination of time, power, angular parameters, and phase spectrum was chosen as the key feature set. MOVENetx64 demonstrated an outstanding performance, with the lowest HOMP prediction errors of 0.6%, 0.17% and 2.28% in the respective driving scenarios. A rigorous comparative analysis was conducted between the proposed model, various standard DL classifiers, and the benchmark multipath classifiers. MOVENetx64 demonstrated superior performance with minimal computational cost, maintaining misclassification rates as low as 1.29%, 0.59% and 3.56% on the respective vehicular use cases. Thus, the comparative analysis justified the capability of the proposed MOVENetx64 in efficiently distinguishing between multipaths across diverse use cases. The redundant time associated with pre-processing of the dataset is eliminated, as the proposed MOVENetx64 renders promising performance on raw, unprocessed and imbalanced data. Hence, MOVENetx64 validates its robustness in efficiently classifying multipaths, and thereby improve vehicular communication by retaining the dominant MPCs and minimising the interferences caused by HOMPs.

Future research shall concentrate on extending the study to Tera-Hertz-based vehicular scenario equipped with unmanned aerial vehicles to enhance the accuracy and flexibility of the DL techniques further.

## Data Availability

The datasets used and analysed during the current study are available from the corresponding author upon reasonable request.
